# An ionophore breaks the multi-drug-resistance of *Acinetobacter baumannii*

**DOI:** 10.15698/mic2022.03.772

**Published:** 2022-02-15

**Authors:** David M.P. De Oliveira, Mark J. Walker

**Affiliations:** 1 The University of Queensland, School of Chemistry and Molecular Biosciences, Australian Infectious Diseases Research Centre, Brisbane, QLD, Australia.

**Keywords:** ionophore, PBT2, antimicrobial resistance, Acinetobacter baumannii, tetracyclines

## Abstract

Within intensive care units, multi-drug resistant *Acinetobacter baumannii* outbreaks are a frequent cause of ventilator-associated pneumonia. During the on-going COVID-19 pandemic, patients who receive ventilator support experience a 2-fold increased risk of mortality when they contract a secondary *A. baumannii* pulmonary infection. In our recent paper (De Oliveira *et al.* (2022), Mbio, doi: 10.1128/mbio.03517-21), we demonstrate that the 8-hydroxquinoline ionophore, PBT2 breaks the resistance of *A. baumannii* to tetracycline class antibiotics. *In vitro*, the combination of PBT2 and zinc with either tetracycline, doxycycline, or tigecycline was shown to be bactericidal against multi-drug-resistant *A. baumannii*, and any resistance that did arise imposed a fitness cost. Using a murine model of pulmonary infection, treatment with PBT2 in combination with tetracycline or tigecycline proved efficacious against multidrug-resistant *A. baumannii*. These findings suggest that PBT2 may find utility as a resistance breaker to rescue the efficacy of tetracycline-class antibiotics commonly employed to treat multi-drug resistant *A. baumannii* infections.

Combating antimicrobial resistance remains a critical global health priority. Over the past decade, the overall trend of bacterial pathogens exhibiting drug resistance has steadily increased. In clinical settings, antibiotic resistance levels of *A. baumannii* are over four-times higher compared to *Klebsiella pneumoniae* and *Pseudomonas aeruginosa*. With the emergence of pan-drug-resistant isolates, last-resort carbapenem and polymyxin class antibiotics are not as effective. The significant decline in antibiotic discovery has paralleled an escalation in antibiotic resistance, highlighting the urgent need for new antibiotic development and complementary therapy.

The hydroxyquinoline ionophore, PBT2, was originally developed as a potential treatment for Alzheimer's and Huntington's disease, progressing to phase 2 human clinical trial with a favourable drug-safety profile. Ionophores such as PBT2, facilitate the passive transport of transition metal ions (e.g. zinc) across biological membranes, altering cellular metal homeostasis. It has been previously demonstrated that PBT2 mediated disruption to metal ion homeostasis enhances antibiotic sensitivity to polymyxin class antibiotics in otherwise resistant strains of multi-drug resistant (MDR) *A. baumannii*. Due to the nephrotoxic side effects of polymyxin class antibiotics, combinational or monotherapy with tetracycline class antibiotics is a preferred treatment option for infection caused by MDR *A. baumannii.* As such we explored the utility of PBT2 to rescue the efficacy of selected tetracycline class antibiotics commonly employed to treat carbapenem- and tetracycline-resistant *A. baumannii* infections.

An increase in endogenous zinc is an important marker of bacterial lung infection. In this study, when combined with zinc, PBT2 was shown to disrupt resistance to tetracycline, doxycycline and tigecycline in four clinically relevant strains of *A. baumannii* with either MDR or extensively drug-resistant profiles. The combination of PBT2 and zinc in the presence of each tetracycline class antibiotic was observed to be bactericidal. We found that in the absence of antibiotic, PBT2 ± zinc increased the membrane permeability of *A. baumannii*. In the presence of tetracycline class antibiotics, PBT2 and zinc exposure caused visible membrane indentations in *A. baumannii* and resulted in bacterial cell rupture.

A key aspect of *A. baumannii* physiology is the propensity to develop rapid resistance. As such we investigated the likelihood of resistance development following treatment with tetracycline class antibiotics, PBT2 and zinc using the MDR *A. baumannii* clinical isolate MS14413. *A. baumannii* MS14413 developed an appreciable increase in resistance to each tetracycline class antibiotic in the presence of PBT2 and zinc. Whole genome sequencing analysis of respective *A. baumannii* MS14413 mutants identified the presence of two major chromosomal changes when compared with the MS14413 reference genome that are associated with resistance to tetracycline class antibiotics: 1) insertion sequence (IS) IS*Aba125* transposition into the TetR-family system regulator gene *adeN,* which regulates the expression of the resistance-nodulation-cell division (RND) pathway, AdeIJK; and 2) IS*Aba1* transposition into the *adeS* gene, encoding the AdeS sensor kinase of the two-component AdeRS regulatory system which regulates the RND AdeABC efflux pump system. Both mutations were shown to accumulate over time and had an additive impact on resistance. Importantly, these respective mutations also imposed a fitness cost as evidenced by reduced growth *in vitro*, and lower survival in the lungs of immunocompetent mice compared with wild-type *A. baumannii* MS14413.

PBT2 has been previously shown to disrupt the dynamic interplay of metal homeostasis in a wide range of Gram-negative and Gram-positive bacteria. In our study, PBT2 increased the cellular content of zinc and copper and reduced magnesium in *A. baumannii* MS14413. Magnesium is known to play a key role in maintaining the structural integrity of the lipopolysaccharide (LPS) outer membrane of Gram-negative bacteria. Through electrostatic interaction, magnesium binds to anionic phosphate groups of the inner core, enabling structural integrity of the outer leaflet. Under magnesium limiting conditions, phosphate groups of the LPS repel each other, decreasing outer membrane stability. Further, disruptions in magnesium dependent bridging of LPS phosphate groups have been shown to cause LPS release from the bacterial cell surface and subsequent bacterial membrane rupture. Here, we hypothesize the PBT2-mediated depletion of cellular magnesium content may mediate the observed increases in membrane permeability and associated membrane defects in *A. baumannii* (**Fig. 1**).

**Figure 1:**
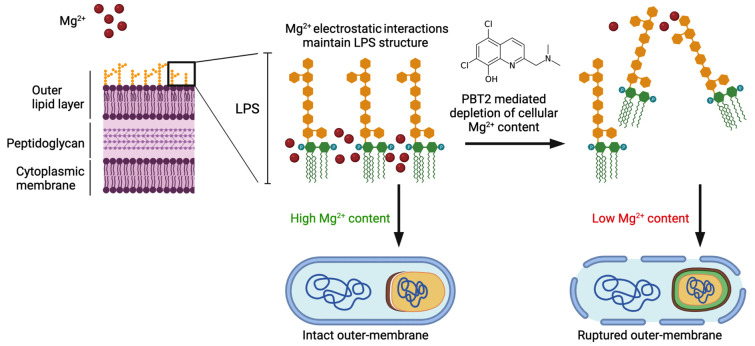
FIGURE 1: Proposed mechanism of PBT2 mediated membrane disruption and bacterial cell rupture. Magnesium binds to anionic phosphate groups of the inner core via electrostatic interaction, enabling structural integrity of the outer leaflet. Disruptions in these important electrostatic cross-link interactions result in lipopolysaccharide (LPS) release and subsequent bacterial membrane rupture. We hypothesize PBT2 mediated dysregulation of bacterial cell metal homeostasis depletes *A. baumannii* of cellular magnesium content resulting in increased membrane permeability and associated membrane defects. Created with Biorender.com.

Transcriptome analysis of *A. baumannii* MS14413 treated with PBT2, zinc and tetracycline identified changes in iron-specific metal stress response systems, and drug efflux systems. Specially, PBT2 led to the upregulation of iron uptake systems, including FeoAB system genes, ferric acinetobactin ABC transporter system genes (*bauC, bauD, and bauE*), and TonB receptor genes (*fpvA* and *pfeA*). Additionally, PBT2 exposure resulted in the upregulation of multidrug efflux resistance nodulation division transport system genes (*adeABC and adeFGH*). These respective transcriptional changes were PBT2 dependent and were not affected by the presence of zinc or tetracycline.

*A. baumannii* is a common cause of ventilator associated pneumonia in intensive care unit-settings. More, several clinical trials (i.e. EURO, REACH, and IMAGINE clinical trials) have demonstrated PBT2 is safe and well-tolerated in humans. As such, we investigated the therapeutic potential of PBT2 to break resistance to of *A. baumannii* MS14413 to tetracycline and tigecycline during a murine model of pulmonary infection. We identified that the combination treatment of either PBT2 and tetracycline, or PBT2 and tigecycline abrogated infection-associated weight loss in mice and resulted in significant 2.5-log and 4-log reductions in the bacterial burden in the lungs, respectively.

Collectively, our study highlights the potential of PBT2 to be used in combination with existing tetracycline class antibiotics for the treatment of pulmonary infection caused by MDR *A. baumannii*. Clinically, one of the greatest unmet needs globally, is in hospital-acquired severe multi-drug resistant Gram-negative bacterial infections – particularly pneumonia caused by *A. baumannii*. Without adequate therapeutic intervention, *A. baumannii* will continue to pose a significant threat to human health.

